# Mechanical Stretch α‐Cyclodextrin Pseudopolyrotaxane Elastomer with Reversible Phosphorescence Behavior

**DOI:** 10.1002/advs.202307777

**Published:** 2024-02-04

**Authors:** Yi Zhang, Yong Chen, Jian‐Qiu Li, Song‐En Liu, Yu Liu

**Affiliations:** ^1^ College of Chemistry State Key Laboratory of Elemento‐Organic Chemistry Nankai University Tianjin 300071 P. R. China

**Keywords:** mechanical response, phosphorescence, pseudopolyrotaxane, supramolecular elastomer

## Abstract

Polyethylene glycol chains in two terminals of the naphthalene functional group are threaded into α‐cyclodextrin cavities to form the pseudopolyrotaxane (NPR), which not only effectively induces the phosphorescence of the naphthalene functional group by the cyclodextrin macrocycle confinement, but also provides interfacial hydrogen bonding assembly function between polyhydroxy groups of cyclodextrin and waterborne polyurethane (WPU) chains to construct elastomers. The introduction of NPR endows the elastomer with enhanced mechanical properties and excellent room temperature phosphorescent (RTP) emission (phosphorescence remains in water, acid, alkali, and organic solvents, even at 160 °C high temperatures). Especially, the reversible mechanically responsive room temperature phosphorescence behavior (phosphorescence intensity increased three times under 200% strain) can be observed in the mechanical stretch and recover process, owing to strain‐induced microstructural changes further inhibiting the non‐radiative transition and the vibration of NPR. Therefore, changing the phosphorescence behavior of supramolecular elastomers through mechanical stretching provides a new approach for supramolecular luminescent materials.

## Graphical abstract

A reversible mechanically stretch enhanced organic room temperature phosphorescent elastomer with excellent mechanical properties and ultrastable afterglow emission is constructed by pseudopolyrotaxane (NPR) and waterborne polyurethane matrix, in which NPR is composed of α‐cyclodextrin and naphthalene modified polyethylene glycol. This strain‐induced microstructural changes driven tunable phosphorescence performance of elastomer provides a new approach for the development of luminescent materials.

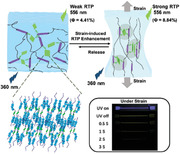

## 1 Introduction

Polyrotaxane (PR) formed by cyclodextrins (CD) threading polymers^[^ ^1^ ^]^ has been extensively exploited for supramolecular soft materials such as adhesives,^[^ ^2^ ^]^ elastomers,^[^ ^3^ ^]^ and hydrogels^[^ ^4^ ^]^ because of its unique topological structure, which can effectively enhance the mechanical properties of materials. The most noteworthy type of PR material is developed by Ito and co‐workers, which has movable cross‐linking sites formed by covalent cross‐linking of CDs and polymers. The pulley effect of CDs effectively dissipates the stress to achieve the high stretchability and reciprocating properties.^[^ ^5^ ^]^ Another emerging type is the use of crystalline domains formed by CDs on PR to provide the non‐covalent cross‐linking for effectively toughening polymer materials.^[^ ^6^ ^]^ Therefore, PR has been introduced into polymer systems as a covalent or non‐covalent cross‐linking agent to obtain desired mechanical properties and has been widely used in the fields of mechanically responsive materials,^[^ ^7^ ^]^ luminescent materials,^[^ ^8^ ^]^ self‐healing materials,^[^ ^9^ ^]^ 3D printing,^[^ ^10^ ^]^ and wearable sensors.^[^ ^11^ ^]^ For example, Ito et al. realized the strain‐induced crystallization in the tough slide‐ring supramolecular hydrogel formed by cross‐linking low coverage PRs, and the reversible slide‐ring cross‐linking sites and force‐induced crystallization regions endowed the hydrogel with the rapid self‐reinforcement, which provided a new idea for the construction of tough hydrogel materials.^[^ ^1a^ ^]^ Ke et al. reported a series of sidechain pseudopolyrotaxane based on polyethylene glycol (PEG) sidechain copolymers and α‐CDs, and by reasonably adjusting the size of the pseudopolyrotaxane crystalline domains and the cross‐linking density of the hydrogels, a sea cucumber simulant with adjustable nano‐to‐macroscale performance was prepared.^[^ ^12^ ^]^ Bao et al. introduced PR into the conductive polymer poly (3,4‐ethylenedioxythiophene): polystyrene sulfonate system to construct a topological supramolecular network, which achieved the high conductivity and the crack‐onset strain simultaneously in the physiological environment for the high spatiotemporal resolution electrophysiological monitoring of biological interfaces.^[^ ^13^ ^]^ We reported the slide‐ring toughened supramolecular mechanoresponsive elastomer by the copolymerization of 2‐(2‐methoxyethoxy) ethyl methacrylate, slide‐ring cross‐linking agent PR, and mechanically responsive group acrylic modified perylene diimides, showing the mechanically induced fluorescence change of elastomer.^[^ ^14^ ^]^ Although PR has been applied to the construction of various soft materials, there are no reports on the mechanical force‐responsive phosphorescence emission systems, to the best of our knowledge. Alternatively, for mechanical force‐responsive phosphorescent materials, the dynamic structure of PR can endow them with enhanced mechanical properties and support the repeated triggering of mechanical force stimulus‐response, achieving the conversion of mechanical force to phosphorescent signals.

**Scheme 1:**
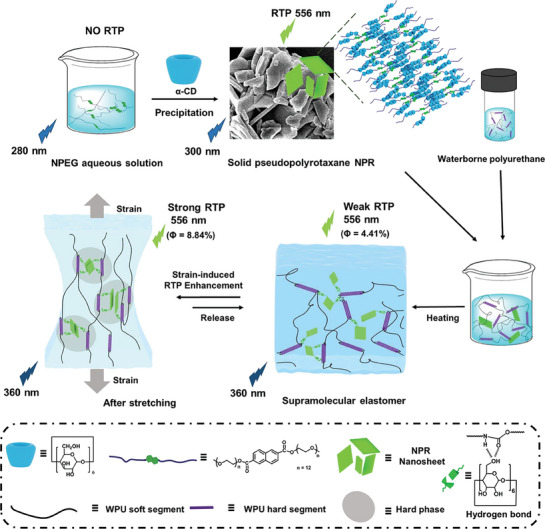
Schematic illustration of mechanically stretched α‐CD pseudopolyrotaxane elastomers with reversible phosphorescence behavior.

Phosphorescence, as the regulatory object of mechanical force response, has the advantages such as large Stokes shift, longer luminescence lifetime, and involvement of triplet states. which has made significant progress in cascading energy transfer,^[^ ^15^ ^]^ two‐photon excitation,^[^ ^16^ ^]^ and stimulus‐responsive luminescence.^[^ ^17^ ^]^ These phosphorescence systems have been successfully applied in the fields of cell imaging,^[^ ^18^ ^]^ anti‐counterfeiting,^[^ ^19^ ^]^ intelligent sensing,^[^ ^20^ ^]^ and lighting devices.^[^ ^21^ ^]^ However, expanding mechanical force‐responsive luminescence to the field of organic phosphorescence still faces some challenges. Mechanical deformation disrupts the interaction between chromophores and polymer matrix, aggravating chromophores vibration and oxygen microenvironment‐mediated non‐radiative quenching of the triplet states, leading to irreversible phosphorescence reduction or quenching in the deformation region.^[^ ^22^ ^]^ Recently, it has been reported that the formation of crystalline phases in a gel matrix can effectively improve the rigidity of the microenvironment and enhance phosphorescence emission.^[^ ^23^ ^]^ Therefore, to achieve mechanically stretch‐responsive supramolecular phosphorescence, the introduction of the strain‐modulated microstructure (reversible microphase separation and strain‐induced crystallization) in room temperature phosphorescent (RTP) materials would be a feasible strategy. Ito et al. reported a strain‐induced crystallization and phase‐separation toughened slide‐ring solid polymer electrolyte, which used slidable hydroxypropyl‐α‐CD ring cross‐linked poly(ethylene oxide) chains as the slidable cross‐linked network and Li salts as solvents.^[^ ^24^ ^]^ Zhang et al. reported the elastomer with a dynamic strain‐induced crystallization network constructed by polyurethane matrix and CD/sodium dodecyl sulfates‐assembled nanosheets, where the crystallization network endowed the elastomer with excellent mechanical properties.^[^ ^25^ ^]^ Herein, we report the supramolecular elastomer based on the waterborne polyurethane (WPU) and the pseudopolyrotaxane NPR formed by α‐CD and the naphthalene‐modified polyethylene glycol (NPEG), which exhibit the reversible mechanically responsive RTP enhancement behavior in the mechanical stretching process (**Scheme** **1**). The introduction of CDs with rich hydroxyl groups not only realizes the long lifetime phosphorescent emission through macrocyclic confinement effect but also enhances mechanical properties by forming hydrogen bonds with WPU chains. The supramolecular elastomer exhibits stable RTP properties (τ = 762.34 ms) and the afterglow emission even in water, high temperatures, and chemical environments (acid, alkali, and organic solvents). Impressively, the reversible phosphorescence emission (phosphorescence intensity increased three times under 200% strain) is observed, as the stretching further suppressed the non‐radiative transition and vibration of NPR. Inspired by its exciting properties, the supramolecular RTP elastomer has been successfully applied to the information security and the encryption.

## 2 Results and Discussion

**Figure 1:**
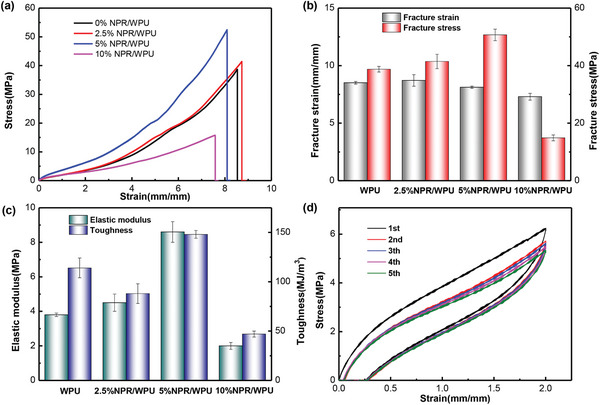
Mechanical performances of supramolecular elastomers. a) Tensile stress‐strain curves. b,c) The fracture stresses, fracture strains, elastic moduli, and toughness of supramolecular elastomers with different NPR masses. d) Cyclic stretching to 200% loading curve of supramolecular elastomer (5% NPR/WPU).

Pseudopolyrotaxane NPR was prepared by α‐CD and NPEG in aqueous solution.^[^ ^14^ ^]^ Based on ^1^H NMR of NPR (Figure S1, Supporting Information), the EG/CD ratio of 2.62 was calculated by comparing the H1 protons of α‐CD at 5.05 ppm (6 H1 protons for 1 α‐CD unit) with the naphthalene protons of NPEG at 8.67 and 8.11 ppm referring to the aromatic region (6 naphthalene protons and 24 EG repeating units for 1 NPEG chain molecule). To study the effect of pseudopolyrotaxane NPR as the non‐covalent cross‐linking agent on supramolecular elastomers, X‐ray diffraction (XRD) and scanning electron microscopy (SEM) experiments of NPR (Figures S2 and S3, Supporting Information) were performed to demonstrate the channel‐type crystal structure with the nanosheet layered morphology. The formation of the NPR crystalline structure is due to the fact that the CDs are threaded onto the PEG by hydrophobic interaction, and adjacent CDs form a channel‐type structure through strong hydrogen bonding interactions among hydroxyl groups, which are further aligned in parallel to form crystals.^[^ ^26^ ^]^ Benefiting from the abundant hydroxyl groups of NPR, the nanosheets can be embedded in the WPU matrix (Figure S4, Supporting Information) through interfacial hydrogen bonding. XRD of the supramolecular elastomer NPR/WPU (Figure S5, Supporting Information) revealed that, as compared with the broad peak of WPU with structure, the elastomer exhibited the new sharp diffraction peak at 20.00°, which corresponded to the channel‐type crystal structure of NPR, indicating that NPR still maintained the crystal structure in the elastomer. To confirm the existence of interfacial hydrogen bonding in NPR/WPU supramolecular elastomers, temperature‐dependent Fourier transform infrared spectroscopy experiment was conducted. The result showed that the peak of the ‐OH group gradually decreased with increasing temperature and shifted from 3368 to 3391 cm^−1^ (Figure S6, Supporting Information), which was attributed to the dissociation of interface hydrogen bonds at the interface between NPR and WPU chains, and the ‐OH peak of supramolecular elastomers at 160 °C was identified with the peak of NPR as shown in Figure S7, Supporting Information. In addition, the bands of N‐H amide I and II in Figure S6, Supporting Information, (black box) showed an overall downward trend. These results indicated the presence of high‐density interfacial hydrogen bonds, which can affect the mechanical properties of elastomer materials. Then the mechanical property characterization results showed that the introduction of NPR can improve the toughness and strength of WPU elastomer (**Figure** **1**a–c). Among the supramolecular elastomers with different masses of NPR (ranging from 2.5 to 10 wt%), 5% NPR/WPU had the best mechanical properties (fracture stress of 50.7 MPa, fracture strain of 812%, toughness of 148 MJ m^−3^, and elastic modulus of 8.6 MPa). Compared to pure WPU, which had the fracture stress of 38.75 MPa, fracture strain of 851%, toughness of 114 MJ m^−3^, and elastic modulus of 3.8 MPa, 5% NPR/WPU showed a significant improvement in mechanical properties. The reciprocating experiments of stretching to 200% (Figure 1d) and 400% (Figure S8a, Supporting Information) indicated that the supramolecular elastomers had good resilience. As shown in Figure S8b, Supporting Information, the NPR/WPU supramolecular elastomer was semitransparent in the original state. As the strain increased, the transparency gradually decreased, and whitening occurred. In contrast, pure WPU elastomers did not show the whitening phenomenon during 400% stretching (Figure S9, Supporting Information). We speculate that this phenomenon is attributed to the aggregation of NPR nanosheets by small strain stretching, resulting in agglomeration with the WPU hard segments and inducing microstructural changes. According to the above experimental results, the mechanical properties of supramolecular elastomers are improved due to the presence of an appropriate amount of NPR, which provides a hydrogen bond cross‐linking site. Additionally, the microstructure of the material as a physical cross‐linking site can effectively dissipate energy. However, an excessive amount of NPR can increase cross‐linking density and even disrupt the interaction between the original polymer chains. This leads to a decrease in the mechanical properties of the elastomer, as demonstrated by the mechanical properties of 10% NPR/WPU. Moreover, the thermal analysis experiment results showed that the introduction of NPR did not significantly change the thermal stability of the WPU elastomer. Both WPU and 5% NPR/WPU exhibited a thermal degradation temperature above 250 °C, with the weight loss rate reaching the maximum at 395 °C (Figure S10, Supporting Information).

**Figure 2:**
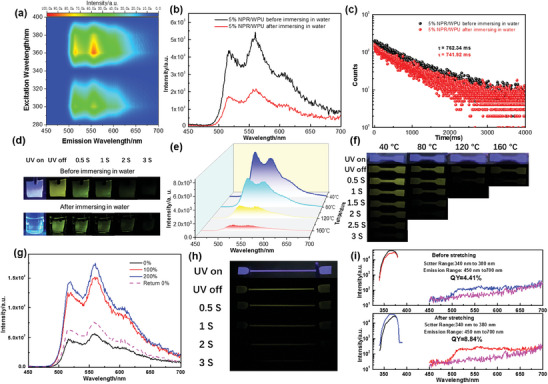
Luminescence performance of NPR/WPU a) Excitation–phosphorescence mapping of NPR/WPU under ambient conditions. b) The phosphorescence emission spectrum (delay = 0.1 ms). c) Time‐resolved photoluminescence decay spectrum at 556 nm of 5% NPR/WPU before and after immersing in water for 30 minutes (*λ*_ex_ = 360 nm). d) Photographs of 5% NPR/WPU before and after immersing in water in different time intervals after excitation at 365 nm. e) The phosphorescence emission spectrum (delay = 0.1 ms) of 5% NPR/WPU at different high temperatures (*λ*_ex_ = 360 nm). f) Photographs of 5% NPR/WPU at different high temperatures in different time intervals after excitation at 365 nm. g) The phosphorescence emission spectrum (delay = 0.1 ms) of 5% NPR/WPU under different strain (*λ*_ex_ = 360 nm). h) Photographs of 5% NPR/WPU at 200% strain in different time intervals after excitation at 365 nm. i) Phosphorescence quantum yield of 5% NPR/WPU before and after stretching.

The introduction of NPR into the WPU network not only enhances the mechanical properties of the elastomer but also endows it with the good luminescent performance. The supramolecular elastomer exhibited the fluorescence emission at 385 nm (τ = 15.89 ns) and the phosphorescence emission at 556 nm (τ = 762.34 ms) (Figures S11 and S12, Supporting Information). It could be seen from **Figure** **2**a that the phosphorescence emission center of the elastomer did not show a shift upon varying the excitation wavelength from 280 to 400 nm. The elastomer displayed the yellow‐green afterglow emission (Figure 2d). The phosphorescence emission spectra of supramolecular elastomers with different NPR contents showed that the phosphorescence intensities of elastomers increased with NPR content (Figure S13, Supporting Information). For subsequent experiments, we selected the 5% NPR/WPU elastomer to study the luminescence behavior due to its optimal mechanical properties. Surprisingly, the elastomer material exhibited the stable phosphorescence behavior, which can prevent the quenching of phosphorescence by water and high temperature. To further investigate this phenomenon, the luminescence behavior of the elastomer was studied under special conditions. After immersing in water, the phosphorescence of the elastomer was not quenched by H_2_O, and the phosphorescence peak remained unchanged despite a decrease in intensity compared to the initial state (Figure 2b), accompanied by the long lifetime of 741.92 ms at 556 nm, and the quantum yield slightly decreased from 4.41% to 4.05% (Figure 2c and Figure S14, Supporting Information). Meanwhile, the high‐temperature luminescence experiment results in Figure 2e indicated that the intensity of phosphorescence decreased with increasing temperature due to the acceleration of non‐radiative decay processes at high temperatures. At the high temperature of 160 °C, the supramolecular elastomer showed the significant phosphorescence with a 57.71 ms lifetime (Figure S15, Supporting Information). High‐temperature spectral testing was not continued due to the elastomer melting at temperatures above 160 °C. The yellow‐green afterglow emission can still be observed by the naked eye (Figure 2d,f, and Video S1, Supporting Information), even when it was immersed in water or at 160 °C. Interestingly, the supramolecular RTP elastomer exhibited the good stability in chemical environments such as trimethylamine (Et_3_N), concentrated HCl, ethanol, and acetone, which maintained a long lifetime (≈ 760 ms) and afterglow emission in these special environments (Figures S16 and S17, Supporting Information). The above experimental results proved that supramolecular elastomers exhibited the ultrastable phosphorescence emission performance in various special environments. To explore the possible mechanism of excellent luminescence, we studied the phosphorescence performance of NPR as an elastomer chromophore under special conditions. First, the ultraviolet (UV) absorption and fluorescence spectra of NPEG showed that it had the fluorescence emission at 388 nm (Figure S18, Supporting Information). Then NPEG interacted with CD to form NPR. NPR emitted the fluorescence emission at 388 nm and a long lifetime phosphorescence at 556 nm with the 3 s yellow‐green afterglow under atmospheric conditions (Figure S19, Supporting Information). After further immersing in water, as shown in Figures S20 and S21, Supporting Information, the phosphorescence emission peaks remained unchanged, accompanied by a decrease in phosphorescence performance (intensity, afterglow time, lifetime, and quantum yield). Figure S22, Supporting Information demonstrated that the high temperature affected the phosphorescence. Specifically, an increase in temperature resulted in a decrease in the intensity, lifetime, and afterglow time of NPR's phosphorescence. NPR still had the phosphorescent emission with a 53.99 ms lifetime and afterglow even at 200 °C (Figure S23, Supporting Information). Additionally, NPR can resist the phosphorescence quenching of strong polar solvents, such as methanol, ethanol, and acetone (Figure S24, Supporting Information). The afterglows of NPR in various special environments were shown in Video S2, Supporting Information. Therefore, the stable phosphorescence behavior of supramolecular elastomer should be attributed to the excellent luminescence of NPR, which is determined by the rich non‐covalent interactions in pseudopolyrotaxane.

To verify the effect of hydrogen bonds on the stable phosphorescence behavior, we placed NPR in the environment of Et_3_Nor concentrated HCl vapor (Figure S25, Supporting Information). The results showed that only proton acids with strong hydrogen bond breaking effect significantly quenched the phosphorescence of NPR surface powder, proving that the stable phosphorescence emission originates from the strong hydrogen bond interactions among CDs. On the other hand, the thermal decomposition temperature of NPR was similar to that of α‐CD, both exceeding 200 °C (Figure S26, Supporting Information). The high‐temperature XRD demonstrated that the crystalline structure of NPR remained stable even at 200 °C, just like that at room temperature (Figure S27, Supporting Information), indicating that the decrease in the phosphorescence intensity and the lifetime caused by the high temperature was due to the accelerated phosphorescence decay by the thermal quenching behavior without crystal structural changes. Then we conducted control experiments on the RTP properties of pseudopolyrotaxane formed by different CDs and their sensitivity to the water environment (Figure S28, Supporting Information). The results showed that the pseudopolyrotaxane formed by β‐CD or γ‐CD with NPEG only exhibited the weak phosphorescence with shorter lifetimes (5.38 ms for NPEG/β‐CD, 6.95 ms for NPEG/γ‐CD at 556 nm) compared to NPR, and their aqueous dispersion exhibited the phosphorescence quenching behavior, with lifetimes reduced to 3.07 and 4.06 ns, respectively. XRD characterization demonstrated that NPEG/β‐CD and NPEG/γ‐CD did not have the regular crystal structure (Figure S29, Supporting Information), indicating that the formation of the crystal structure was crucial for the stable phosphorescence emission. All the above experimental results have confirmed that the rich hydrogen bonds and the channel‐type crystalline structure of NPR are key factors for stabilizing RTP luminescence. These can ensure intermolecular interactions confine chromophores to a rigid environment, effectively limit chromophore vibration and motion, reduce non‐radiative transitions, and weaken the quenching of phosphorescence by external environments (such as high temperature, H_2_O, and O_2_). The elastomer obtained by only mixing NPEG into the WPU matrix did not show the phosphorescence emission, which proved that the confinement effect of CD played a decisive role in the phosphorescence emission of elastomer (Figure S30, Supporting Information). Therefore, the outstanding environmental stability of supramolecular elastomers can be attributed to the synergistic effect of CD confinement and polymer networks. Multiple intermolecular interactions can effectively limit the movement of phosphorescent chromophores and inhibit the non‐radiative decay, and the network structure provides a rigid environment to stabilize the triplet state, avoiding the intrusion of quenching agents and reducing the RTP quenching.

**Figure 3:**
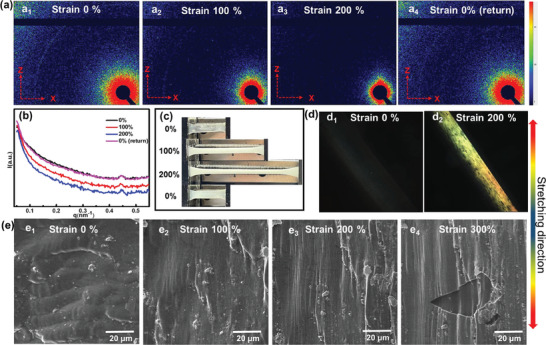
a) In situ tensile 2D SAXS images of 5% NPR/WPU during stretching and reciprocating process. b) 1D scattering profiles of different strain. c) Photographs of 5% NPR/WPU after stretching and reciprocating. d) POM images of 5% NPR/WPU under 0% and 200% tensile strains. e) SEM images of 5% NPR/WPU under different strains.

Most importantly, the supramolecular elastomer exhibited stretch‐induced phosphorescence enhancement. The phosphorescence spectra of supramolecular elastomers under strain indicated that the phosphorescence intensity of the elastomer increased by approximately three times at the 200% strain compared to the original state (Figure 2g), accompanied by the lifetime change from 762.34 to 774.57 ms (Figure S31, Supporting Information), and the quantum yield increased from 4.41% to 8.84% (Figure 2i). The afterglow photos of the elastomer under the strain state showed that the phosphorescence intensity of the stretched part was significantly stronger than that of the unstressed (Figure 2h and Video S1, Supporting Information). To gain more insights into this unique luminescence behavior, the in situ uniaxial tensile 2D small‐angle X‐ray scattering (SAXS) was conducted to reveal the microstructural changes of the elastomer under mechanical force (**Figure** **3**a). The obtained results showed that the 2D SAXS image exhibited a significant change from circular to rhombic with increasing strain, and the scattering intensity on the z‐axis increased, indicating that the elastomer exhibited the orientated structure under 200% strain. The polarization optical microscopy (POM) images showed that the elastomer exhibited significant birefringence at 200% strain (Figure 3d_2_) compared to the original state without birefringence (Figure 3d_1_), which suggested the formation of anisotropic microstructure induced by strain. SEM images of supramolecular elastomers under different strains showed that the elastomer formed the increasingly obvious aligned morphology and the orientated structure along the direction of external forces as the strain increased (Figure 3e). Interestingly, we captured a cracked morphology at 300% strain (Figure 3e_4_), which clearly showed that this oriented structure was not only manifested in the external morphology but also in the internal structure of the elastomer. The atomic force microscope (AFM) and XRD experiments results of the elastomer under different strains (Figures S32 and S33, Supporting Information) showed that the slight enhancement of the shoulder peak at 13.1° in XRD may be due to the crystallization formed by hard segment of WPU with NPR through hydrogen bonding, and then, AFM images showed phase separation. One possible explanation was that the strain‐induced phase separation led to the strain‐enhanced mechanical properties and phosphorescence behaviors, although we did not have other direct evidence. After unloading, the 1D SAXS profile and 2D SAXS image of the elastomer returned to their original state (Figure 3b,a_4_), as well as the disappearance of stretch‐induced whitening phenomenon and the restoration of transparency (Figure 3c). The reversible microstructure determined the reversible phosphorescence emission. After 5 cycles of reciprocating stretching, the phosphorescence intensity of the elastomer can still increase by two times under 200% strain (Figure S34, Supporting Information). Due to the irreversible recovery of the strain‐induced whitening phenomenon when stretched to 400% (Figure S8, Supporting Information), the strain‐induced enhanced phosphorescence emission behavior was only studied at 200% strain. Therefore, we speculate that NPR can promote microstructural changes of elastomers, which determine the reversible modulation properties of elastomeric phosphorescence emission. Under strain, the directional arrangement of polymer chains promoted the close packing of NPR with the hard segment of WPU, thus changing the microenvironment of NPR, preventing the non‐radiative transition of NPR, and realizing the mechanical force‐enhanced phosphorescence behavior.

**Figure 4:**
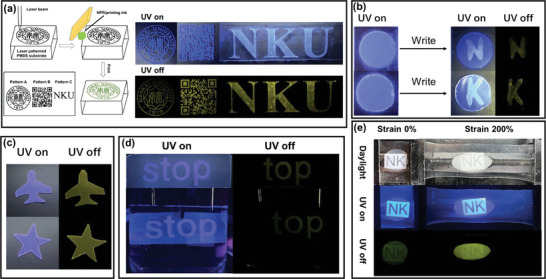
a) Laser engraving PDMS matrix patterning scheme and information storage process by printing NPR/ink onto the PMDS substrate under 365 nm UV light on and off. b) Writable RTP hydrogel for information storage under 365 nm UV light on and off. c) Photographs of NPR/WPU RTP elastomer with different shapes (aircraft and pentagram) by tailoring processing under 365 nm UV light on and off. d) Data encryption process by fixing tailored luminescence elastomer in WPU elastomer in the atmospheric and water environment (pattern “s”: NPEG/WPU samples; pattern “top”: NPR/WPU samples). e) Luminescence photographs of elastomers at different strains taken under daylight, 365 nm UV light on and off (pattern circular: NPR/WPU samples).

Inspired by the unique RTP properties, NPR and NPR/WPU elastomers have been explored for information storage and encryption. **Figure** **4**a showed the information pattern grooves carved by laser on a polydimethylsiloxane (PDMS) substrate and then printed with 10% NPR/printing ink into the pattern grooves. In this way, the emblem of Nankai University, the QR code, and the letter “NKU” representing Nankai University were stored on PDMS film. This allowed a phone to scan QR code of different colors in both UV on and UV off states, providing access to web information. As a result, supramolecular materials can be successfully applied in information storage. Utilizing the stable RTP emission property of NPR in aqueous solution, the polyethylene glycol hydrogel doped with NPEG was prepared through free radical copolymerization as a functional paper for information storage (Figure 4b and Video S3, Supporting Information). The hydrogel showed the blue fluorescence of NPEG, and messages can be written with saturated α‐CDs aqueous solution as ink. After the UV light was removed, the hydrogel only showed the afterglow image of the written information, which was due to the formation of NPR with stable afterglow emission through hydrophobic interaction in the hydrogel. NPR/WPU elastomer can be programmed and processed into various shapes with afterglow emissions, such as aircraft and pentagram, as shown in Figure 4c and Video S3, Supporting Information. Then we processed the NPEG/WPU elastomer into the letter “s” and the NPR/WPU elastomer into the letter “t,” “o,” and “p.” The letters were arranged and embedded into the WPU matrix to prepare a whole block of hybrid elastomer (Figure 4d and Video S3, Supporting Information). Whether in the atmosphere environment or immersed in the aqueous solution, the hybrid elastomer displayed the blue fluorescence message “stop” under UV irradiation. After removing the UV light, the luminescence of the letter “s” immediately disappeared as the absence of afterglow emission from NPEG/WPU, the letter “top” remained visible with the stable afterglow emission of the NPR/WPU, thus obtaining the correct information “top.” This indicates that the mixed elastomer can achieve information encryption in special underwater environments. Due to the same polymer skeleton, the hybrid elastomer with strong interfacial adhesion was obtained by embedding circular NPR/WPU elastomers into the WPU polymer matrix (Figure 4e and Video S3, Supporting Information). The tensile force at the terminal of the WPU matrix caused the deformation of the embedded NPR/WPU elastomer. Under both daylight and UV irradiation, the embedded elastomer had high transparency in the original state, allowing the “NK” on the paper below to be seen. After stretching, the transparency decreased, and the observed “NK” was unclear. However, the decrease in transparency did not affect the more obvious display of “NK” under afterglow illumination after the UV off, as the phosphorescence of the elastomer was significantly enhanced after stretching. Moreover, changes in the shape and transmittance of the embedded elastomer can be used to detect the tensile direction and local strain of the embedded elastomer. The above experiments successfully applied elastomer with excellent RTP to information storage and encryption under special conditions.

## 3 Conclusion

In summary, the novel organic RTP supramolecular elastomer with mechanically responsive phosphorescence enhancement properties was constructed by WPU matrix and cyclodextrin pseudopolyrotaxane NPR formed by α‐CD and NPEGthrough host‐guest interactions. The elastomers not only exhibit excellent phosphorescence properties, which can emit afterglow in water, acid, alkali, organic solvents, and even high‐temperature conditions, but also show tunable phosphorescence behavior driven by mechanical stretching. The multiple interactions in the elastomer, such as CD confinement and interfacial hydrogen bond, effectively stabilize the triplet state, limit the vibration of the chromophore, and promote stable phosphorescence emission in special environments. The reversible strain‐induced microstructural changes endow elastomers with enhanced mechanical properties and tunable phosphorescence emission properties due to effective dissipation of energy and further limitation of the chromophore non‐radiative transitions. Utilizing unique RTP performance, this supramolecular elastomer can be applied to anti‐counterfeiting, display, and information encryption in special environments.

## Conflict of Interest

The authors declare no conflict of interest.

## Supporting information

Supporting Information

Supplemental Video 1

Supplemental Video 2

Supplemental Video 3

## Data Availability

The data that support the findings of this study are available from the corresponding author upon reasonable request.
