# Supplementary material for: Mechanical Stretch α‐Cyclodextrin Pseudopolyrotaxane Elastomer with Reversible Phosphorescence Behavior
Source: Adv Sci (Weinh). 2024 Feb 4;11(14):2307777. doi: 10.1002/advs.202307777 (PMC11005743; doi:10.1002/advs.202307777)
Supplement: Supplementary file 1 — Supporting Information [file ADVS-11-2307777-s001.pdf]

ADVANCED SCIENCE
Open Access

# Supporting Information

for *Adv. Sci.*, DOI 10.1002/advs.202307777

Mechanical Stretch $\alpha$-Cyclodextrin Pseudopolyrotaxane Elastomer with Reversible Phosphorescence Behavior

*Yi Zhang, Yong Chen, Jian-Qiu Li, Song-En Liu and Yu Liu**

Supporting Information

**Mechanical Stretch α-Cyclodextrin Pseudopolyrotaxane Elastomer with Reversible Phosphorescence Behavior**

*Yi Zhang, Yong Chen, Jian-Qiu Li, Song-En Liu and Yu Liu**

Y. Zhang, Prof. Y. Chen, Q. Li, S. Liu, Prof. Yu Liu
College of Chemistry
State Key Laboratory of Elemento-Organic Chemistry
Nankai University, Tianjin 300071, P. R. China
E-mail: yuliu@nankai.edu.cn

## Experimental section

### Materials

α-CD (98%) is purchased from Macklin. NPR is synthesized according to our report[1]. Waterborne PU was purchased from Bayer Co., LTD (China). All the reagents and solvents were commercially available and used as received unless otherwise stated.

### Synthesis of NPR/WPU Elastomers

6g of waterborne polyurethane (60 wt.%) and different masses of NPR (16.6 wt.% aqueous solution) were stirred for 30 minutes to obtain mixed suspension. Then, the suspension was coated on a glass substrate and heated in the oven at 60 °C for 24 hours to obtain the elastomers.

### Preparation of writable PEG hydrogel

850 mg of polyethylene glycol methyl methacrylate (average Mn = 2000, 50 wt% in $H_2O$, Aldrich), 150 mg of polyethylene glycol methyl methacrylate (average Mn = 700, Aldrich), and 40 mg of NPEG were dissolved in 1ml aqueous solution. 30 mg of

$K_2(SO_4)_2$ was added to the solution under stirring until dissolved, then 3 μL of N, N, N, N-tetramethylethylenediamine was added. The solution was poured into the mold and polymerized at 40 °C for 30 min to obtain hydrogel.

**Instrumentation and methods**

NMR measurements were performed on a Bruker AV400 instrument. The solid-state UV/vis spectrum was acquired on a Varian Cary 5000 instrument. Photoluminescence spectra, phosphorescence lifetimes and quantum yields were measured on an FLS5 instrument (Edinburg Instruments, Livingstone, UK). High temperature delay spectroscopy and phosphorescence lifetime were measured on an FS920P instrument (Edinburg Instruments, Livingstone, UK). Tensile tests were conducted on a universal tensile machine (UTM, Shenzhen Suns Technology Stock Co. Ltd) with a stretching rate of 50 mm $min^{-1}$ for uniaxial tensile measurements and 50 mm $min^{-1}$ for cyclic loading–unloading tests at room temperature for more than three times. The elastomers were cut into GB/T-528 IV dumbbell-shaped slices for testing at 25 °C. Thermogravimetric analysis (TGA) experiment was performed on a NETZSCH METTLER-TOLEDO TG209/DSC204 under ambient conditions from 25 to 600°C with a heating rate of 10 °C $min^{-1}$. In situ stretching SAXS measurement was obtained from SAXS apparatus Xeuss 2.0 and a Rayonix MX225-HE CCD X-ray detector was used. AFM images were acquired on Bruker Dimension Icon AFM. Polarized optical microscopy (POM) images were observed on polarized optical microscope (Olympus BX51TRF). In situ SEM experiment was carried out on the Phenom XL G2 microscope operating at 10 KV. Temperature-dependent FTIR

spectroscopy was conducted on a Bruker TENSOR II. The test condition was heating from 30°C to 160°C at 10°C • $min^{-1}$. XRD patterns were analyzed on a Rigaku SmartLab 9KW instrument.

**Supplemental figures**

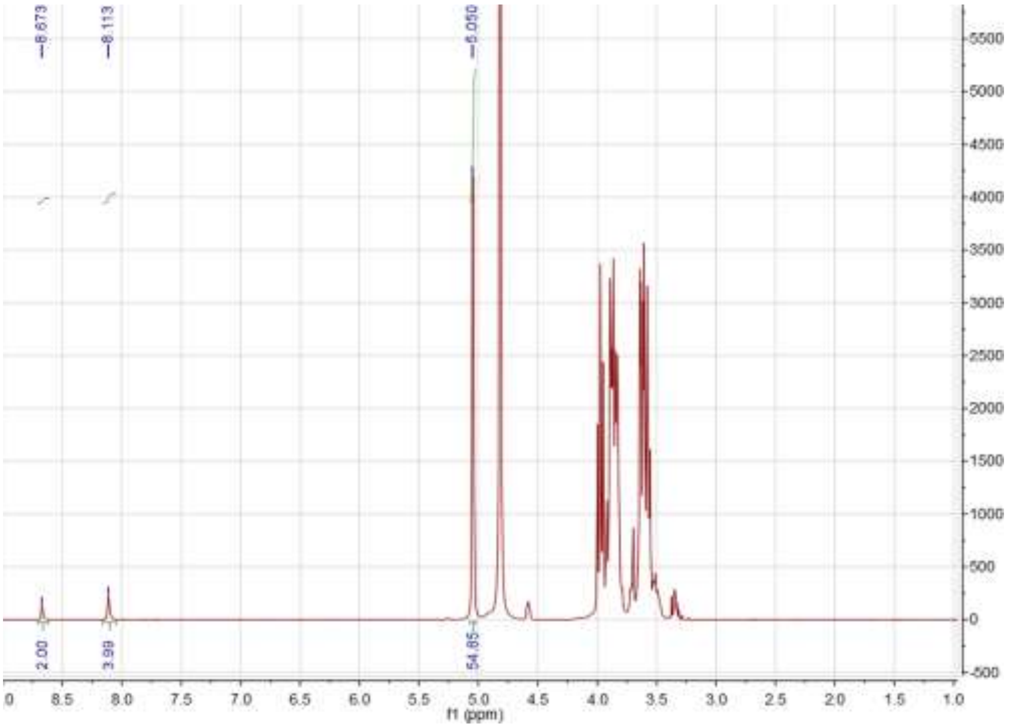


Figure S1. $^{1}H$ NMR of NPR (400 MHz, $D_2O$, 25°C)

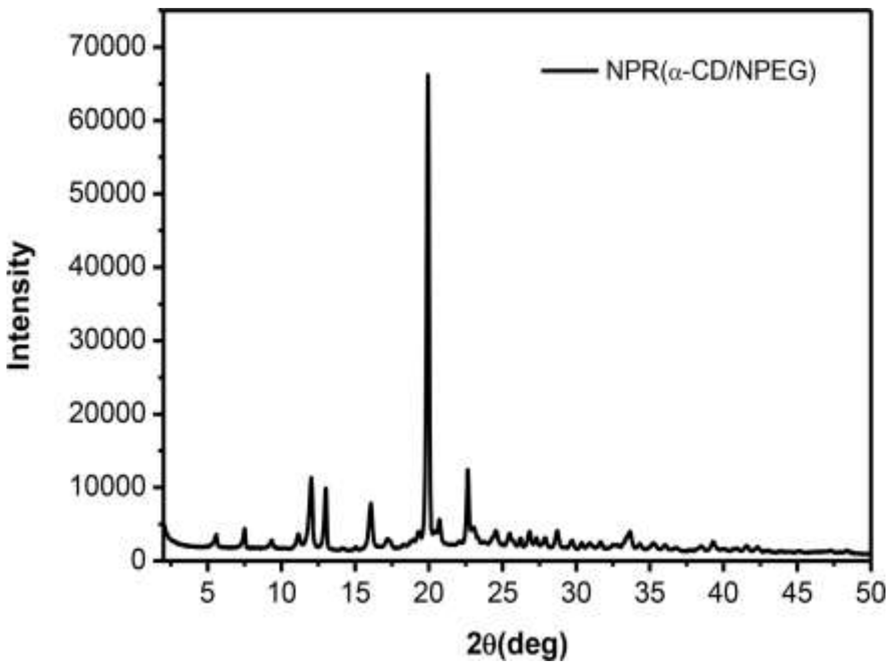


Figure S2. XRD spectrum of NPR.

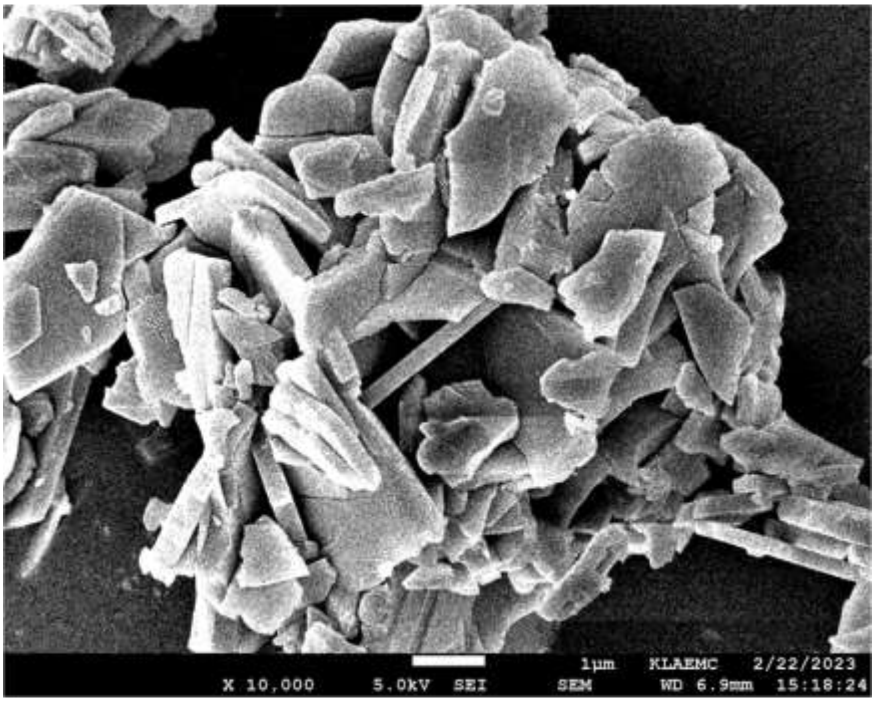


Figure S3. SEM image of NPR.

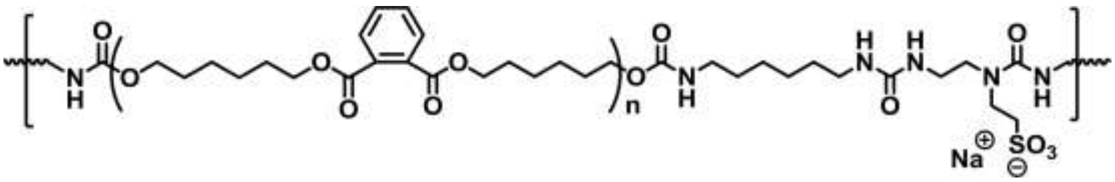

Figure S4. The main structure of the WPU chain.

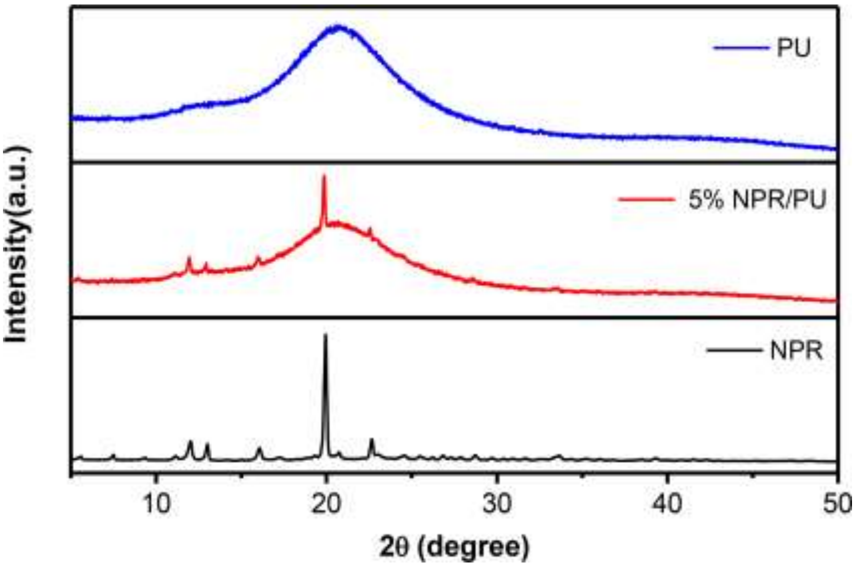


Figure S5. XRD spectrum of PU, 5% NPR/PU, and NPR.

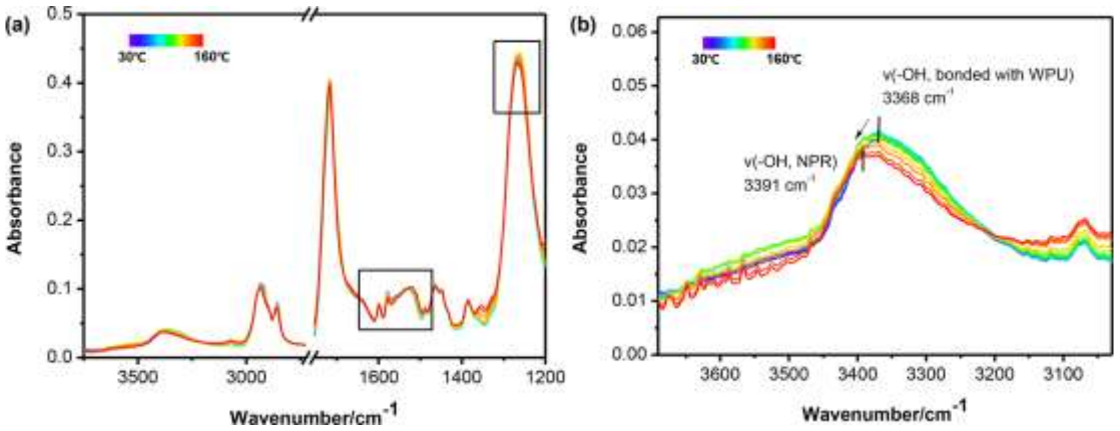


Figure S6. Temperature-dependent FTIR spectra of 10 wt % NPR/WPU supramolecular elastomer upon heating from 30 °C-160 °C.

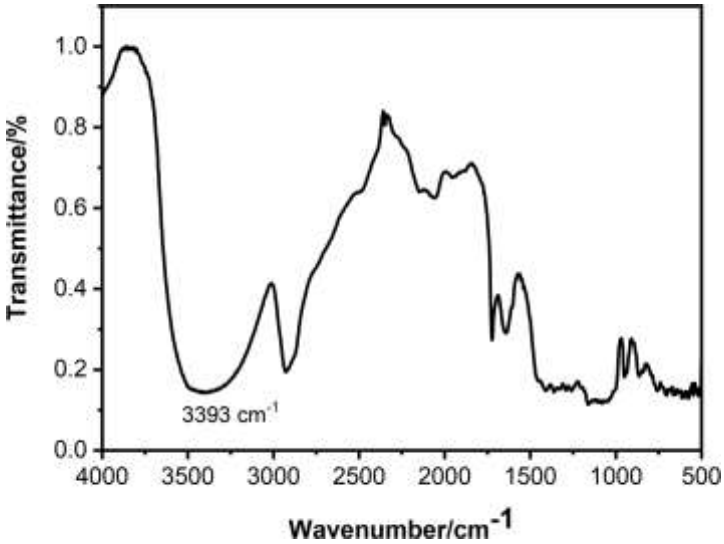

Figure S7. FTIR spectrum of NPR at 30 °C.

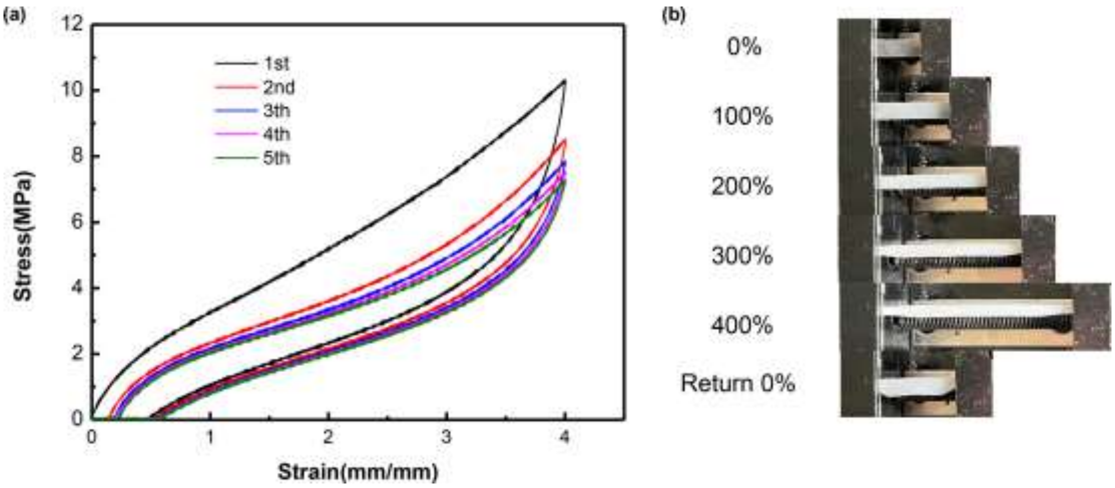


Figure S8. (a) Cyclic stretching to 400% loading curve of supramolecular elastomer 5% NPR/WPU. (b) Photographs of the 5% NPR/WPU with different strain.

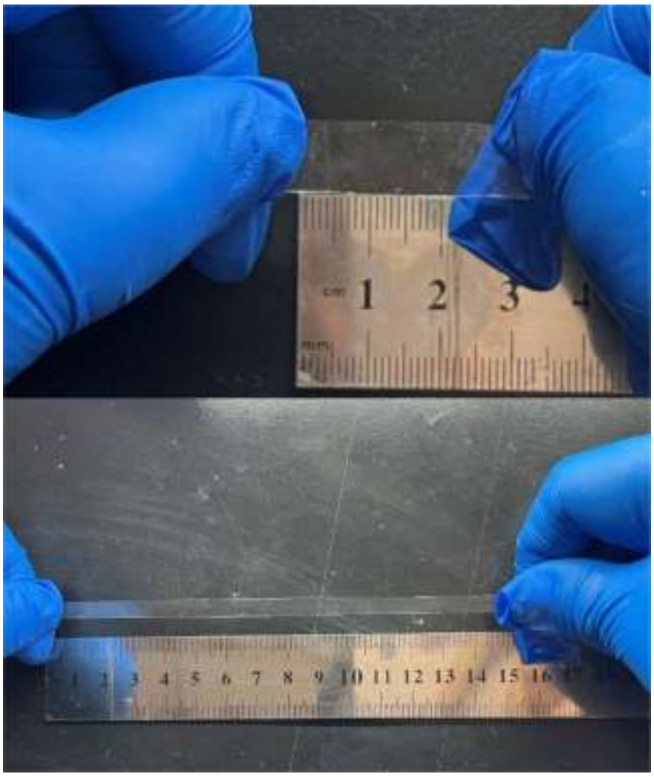

Figure S9. Photographs of the WPU with different strain.

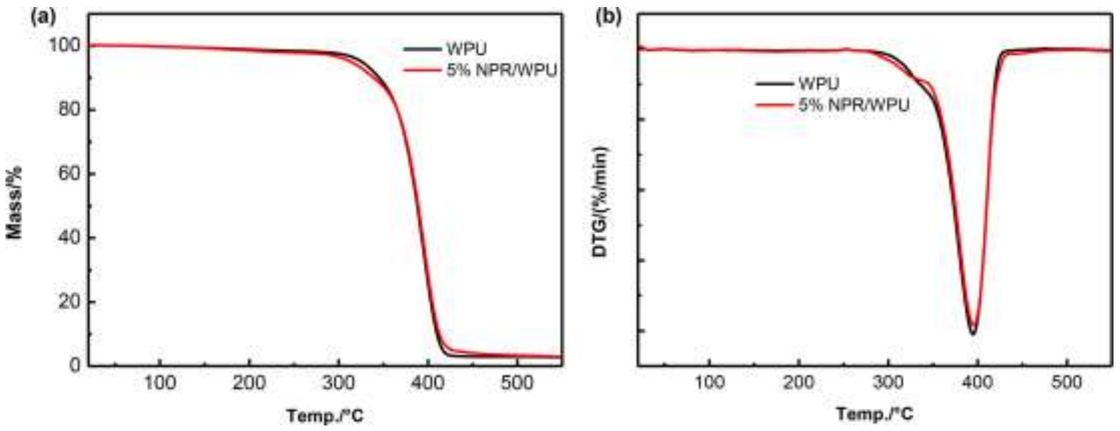


Figure S10. (a) TGA curves and (b) DTG curves of WPU and 5% NPR/WPU.

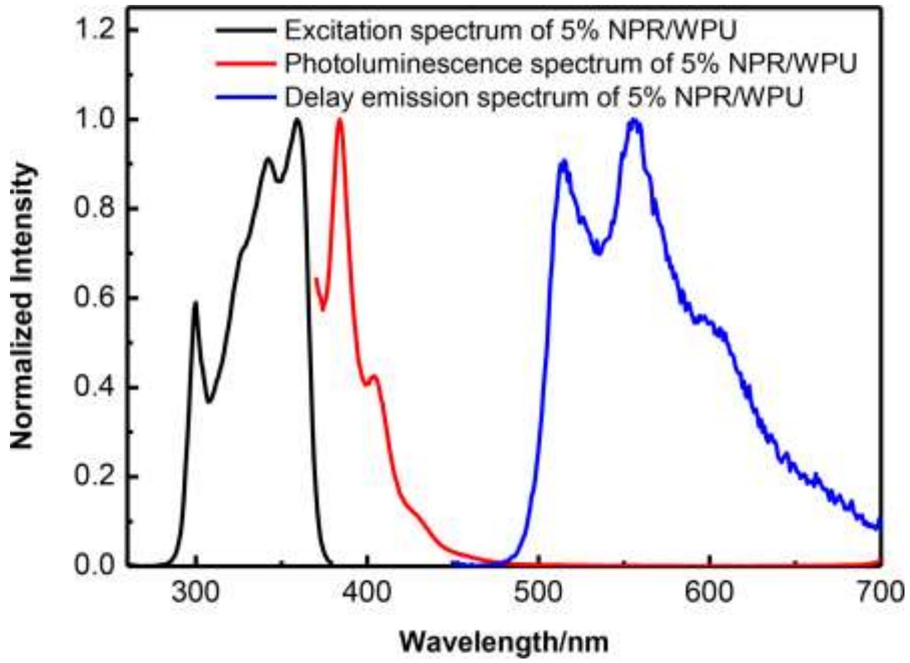


Figure S11. Normalized excitation spectrum, photoluminescence spectrum and the phosphorescence emission spectrum (delay = 0.1 ms) of 5% NPR/WPU.

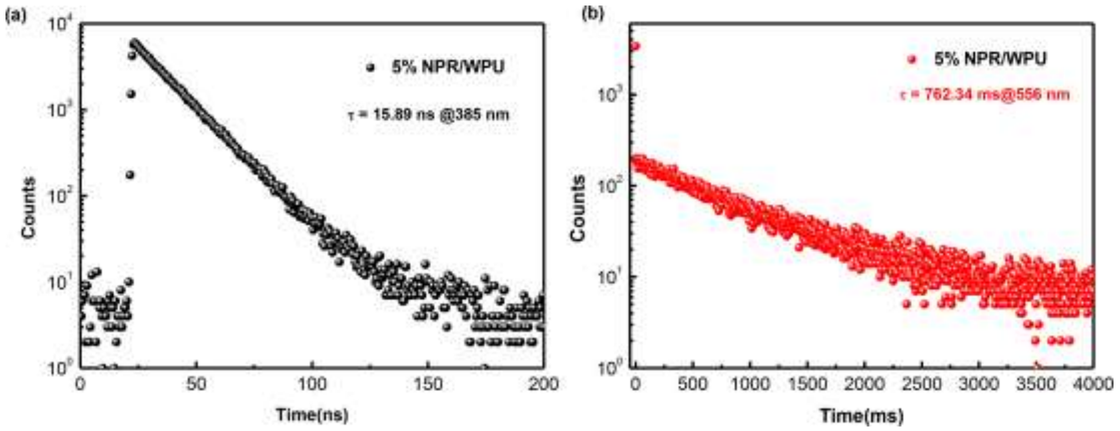


Figure S12. (a) Time-resolved photoluminescence decay spectrum of 5% NPR/WPU

at 385 nm (b) Time-resolved photoluminescence decay spectrum of 5% NPR/WPU at 556 nm.

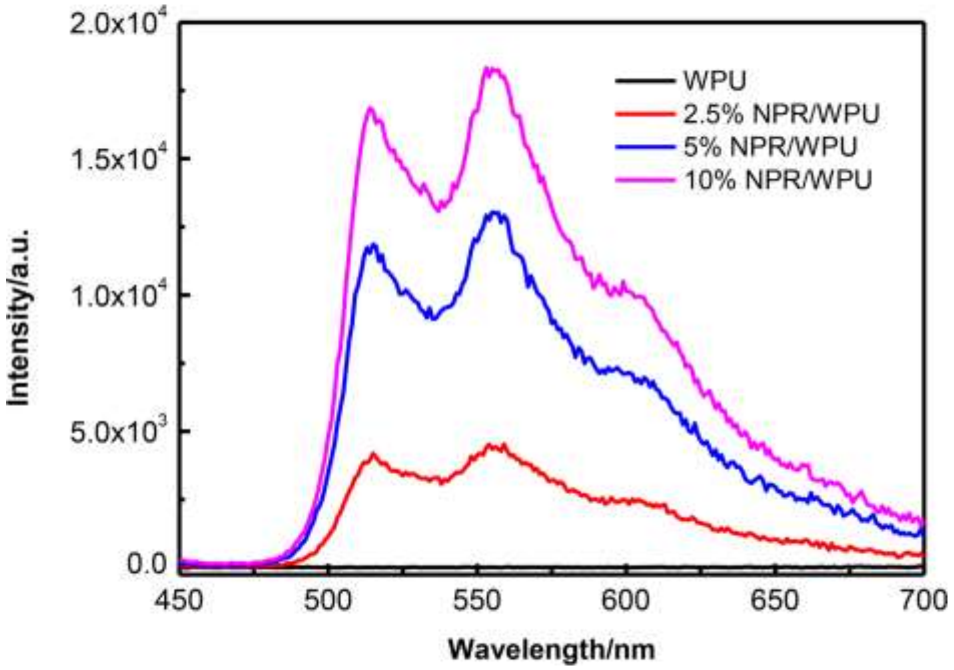


Figure S13. The phosphorescence emission spectrum (delay = 0.1 ms) of elastomers with different NPR contents.

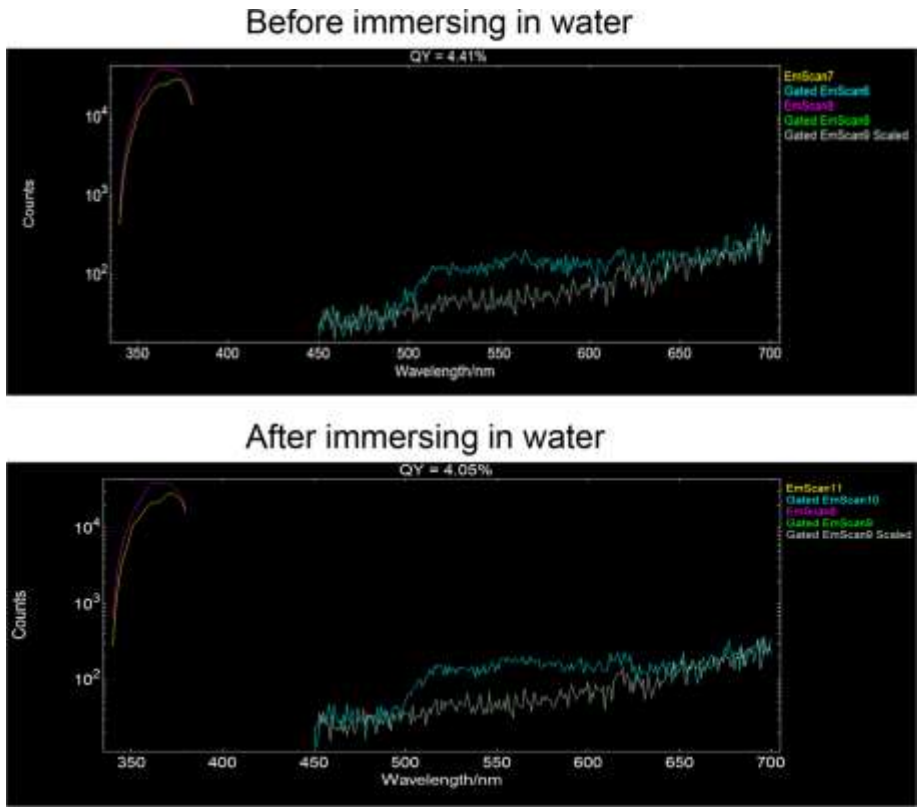


Figure S14. The phosphorescence quantum yield of 5% NPR/WPU before and after soaking in water.

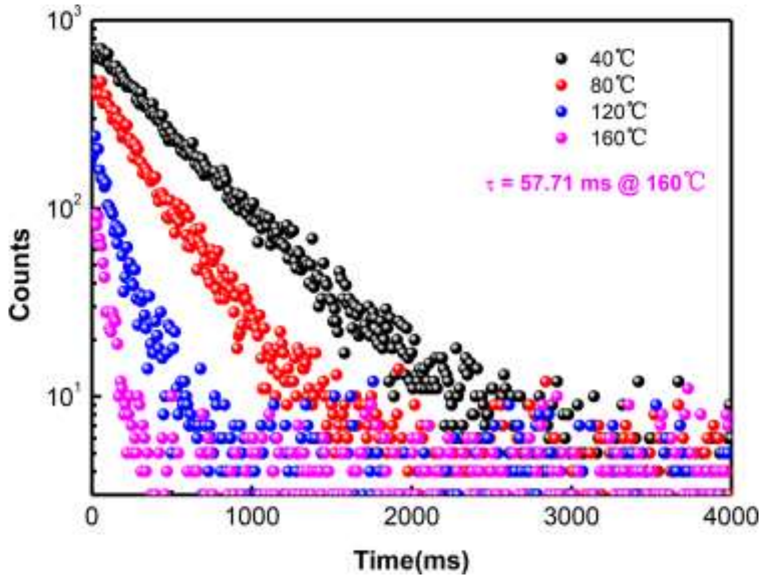


Figure S15. Time-resolved photoluminescence decay spectra of 5% NPR/WPU at different high temperatures.

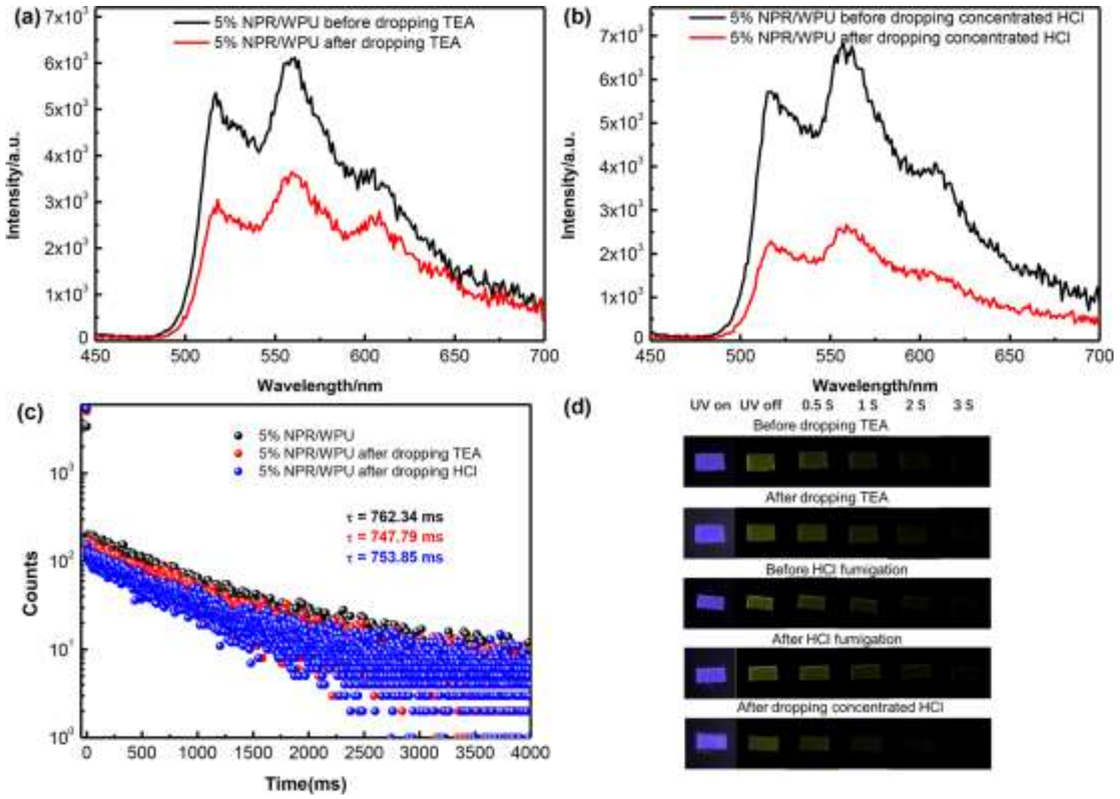


Figure S16. The phosphorescence emission spectra (delay = 0.1 ms) of 5% NPR/WPU (a) before and after dropping TEA (b) before and after dropping concentrated HCl. (c) Time-resolved photoluminescence decay spectra at 556 nm before and after dropping TEA and concentrated HCl. ($\lambda_{ex}$ = 360 nm). (b) Photographs of 5% NPR/WPU before and after dropping TEA, HCl vapor fumigation and concentrated HCl in different time intervals after excitation at 365 nm.

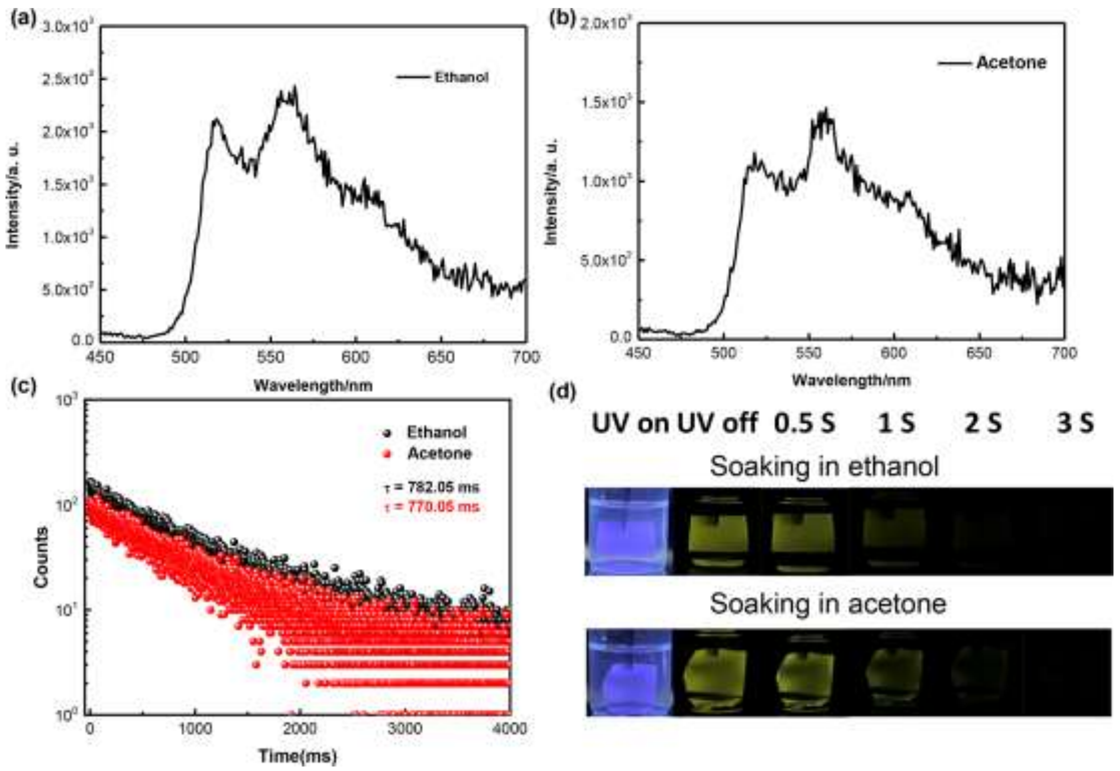


Figure S17. (a) The phosphorescence emission spectrum (delay = 0.1 ms) of 5% NPR/WPU after soaking in ethanol for 30 minutes. (b) The phosphorescence emission spectrum (delay = 0.1 ms) of 5% NPR/WPU after soaking in acetone for 30 minutes. (c) Time-resolved photoluminescence decay spectra at 556 nm of 5% NPR/WPU after soaking in ethanol and acetone for 30 minutes. (d) Afterglow photographs of 5% NPR/WPU after soaking in ethanol and acetone for 30 minutes.

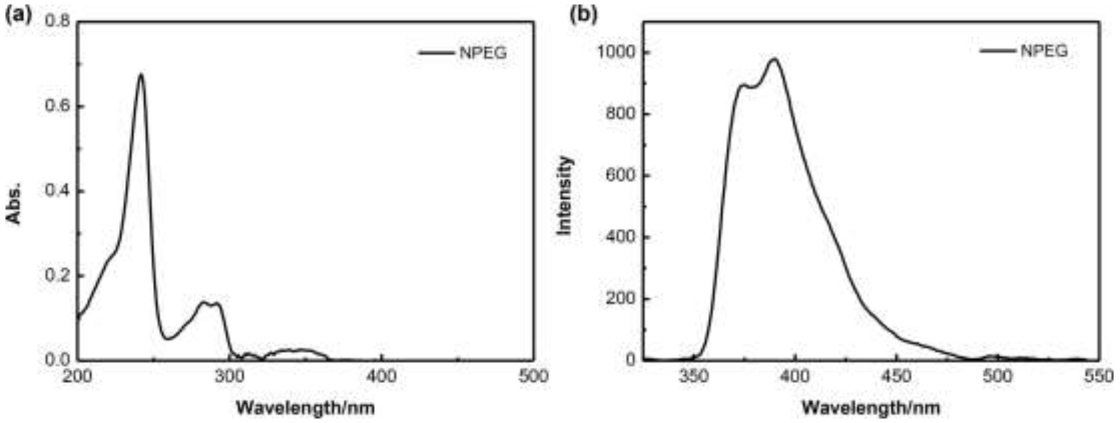


Figure S18. (a) UV/vis spectrum of NPEG (0.01 mM). (b) Fluorescent spectrum of NPEG (0.01 mM) in water ($\lambda_{ex}$=280 nm).

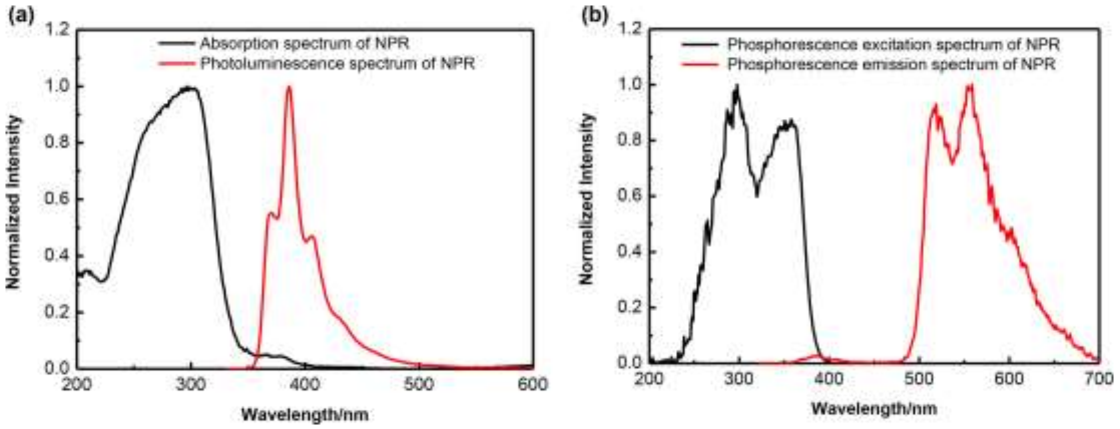


Figure S19. (a) Normalized UV absorption spectrum and photoluminescence spectrum of NPR under the solid powder. (b) Normalized phosphorescence excitation spectrum and emission spectrum of NPR under the solid powder.

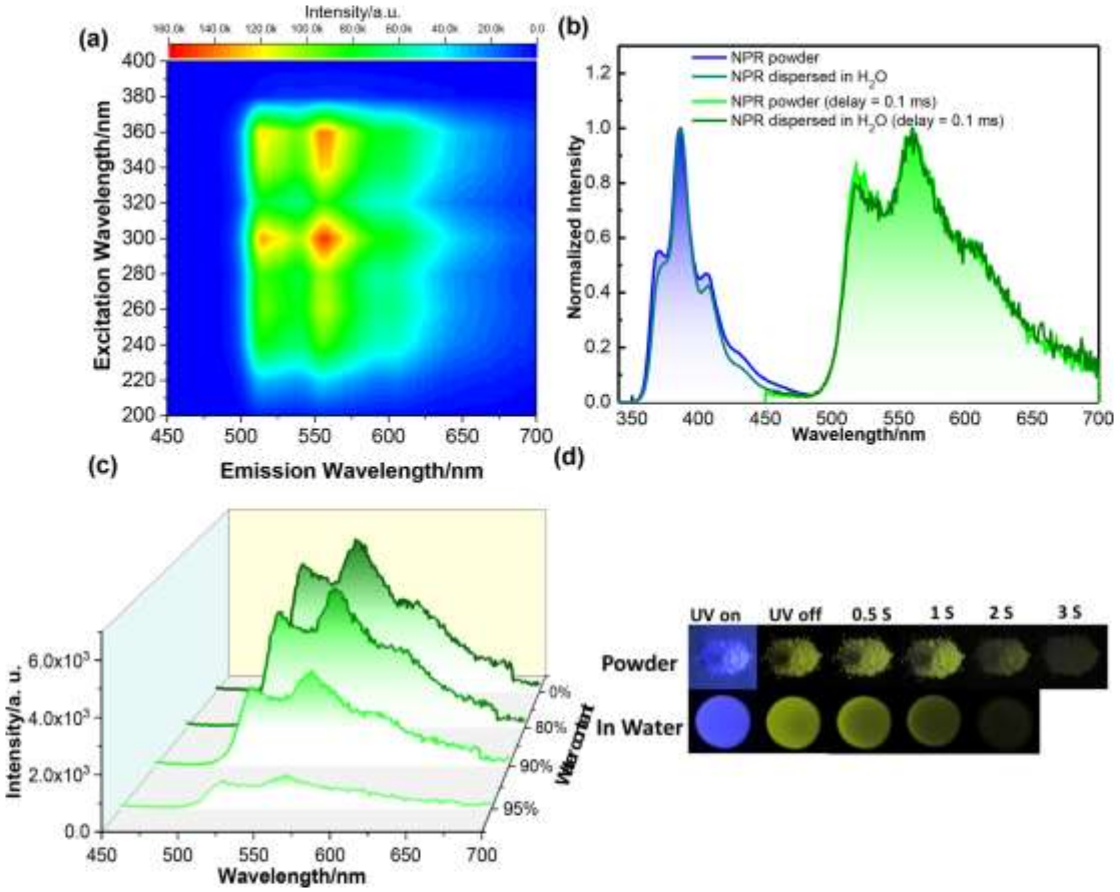


Figure S20. Luminescence performance of NPR (a) Excitation–phosphorescence mapping of NPR excited under ambient conditions. (b) The photoluminescence spectra and phosphorescence emission spectra (delay = 0.1 ms) of NPR in the solid powder and in the 90 wt% $H_2O$ ($\lambda_{ex}$ = 300 nm). (c) The phosphorescence emission spectra (delay = 0.1 ms) of NPR dispersed in different amounts of $H_2O$ ($\lambda_{ex}$ = 300 nm).

(d) Photographs of NPR in the solid powder and in the 90 wt% $H_2O$ in different time intervals after excitation at 303 nm.

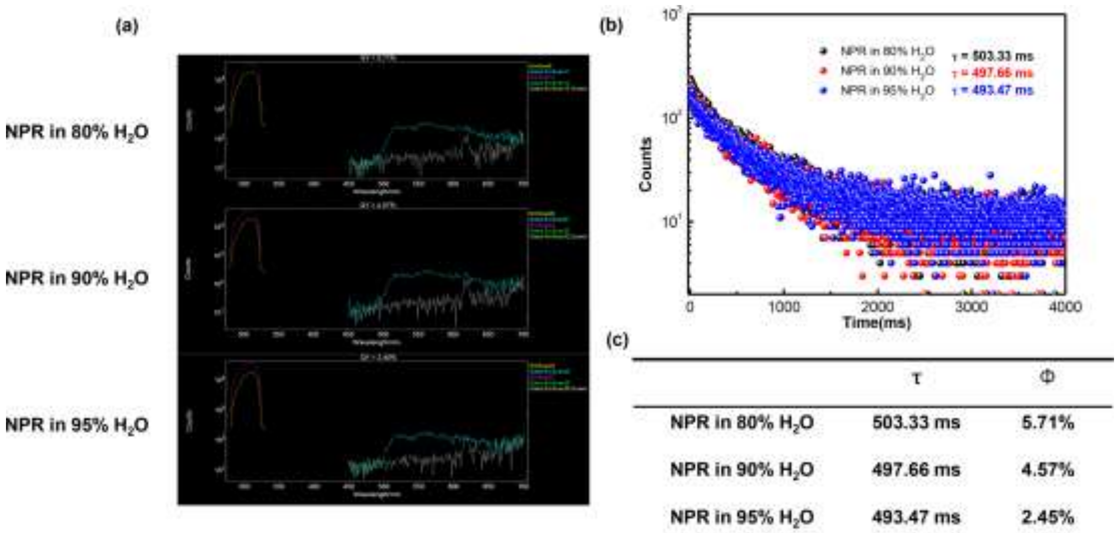


(c)

| | τ | Φ |
|---|---|---|
| NPR in 80% $H_2O$ | 503.33 ms | 5.71% |
| NPR in 90% $H_2O$ | 497.66 ms | 4.57% |
| NPR in 95% $H_2O$ | 493.47 ms | 2.45% |

Figure S21. (a)The phosphorescence quantum yield (b) Time-resolved photoluminescence decay spectra of NPR in different water contents. (c) Table of NPR Lifetime and phosphorescence quantum yield of NPR in different water contents.

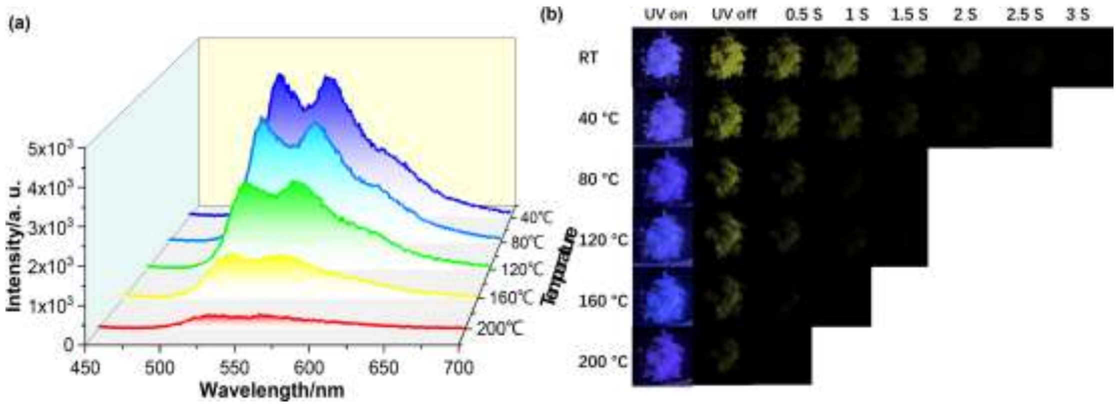


Figure S22. (a) The phosphorescence emission spectra (delay = 0.1 ms) of NPR at different high temperatures ($\lambda_{ex}$ = 300 nm). (b) Photographs of NPR at different high temperatures in different time intervals after excitation at 303 nm.

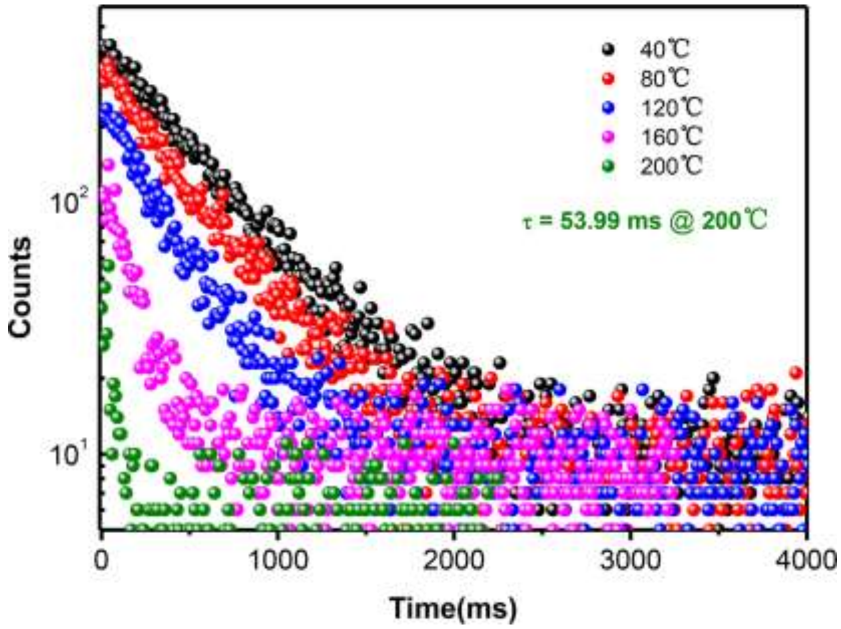


Figure S23. Time-resolved photoluminescence decay spectra of NPR at different high temperatures.

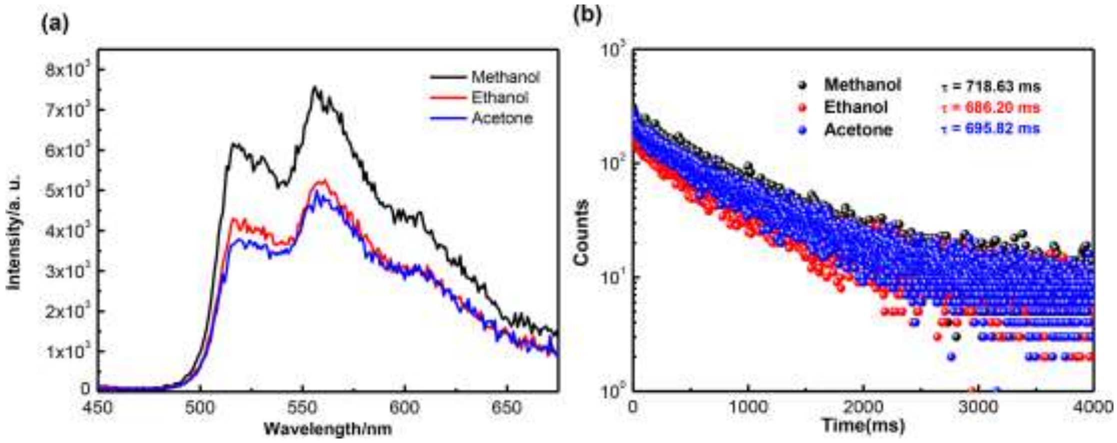


Figure S24. (a)The phosphorescence emission spectra (delay = 0.1 ms) (b) Time-resolved photoluminescence decay spectra of NPR in different polar solvents.

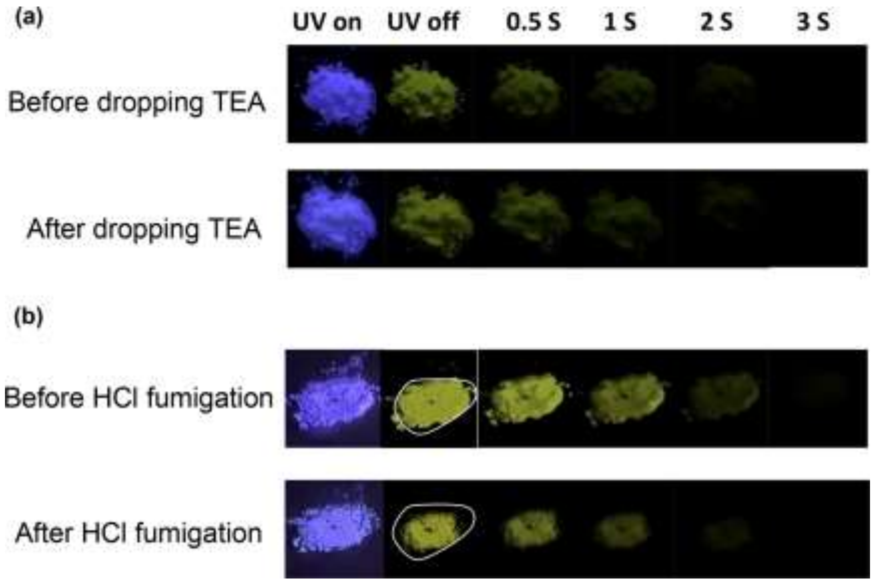


Figure S25. Photographs of NPR before and after (a) dropping TEA (b) HCl vapor fumigation.

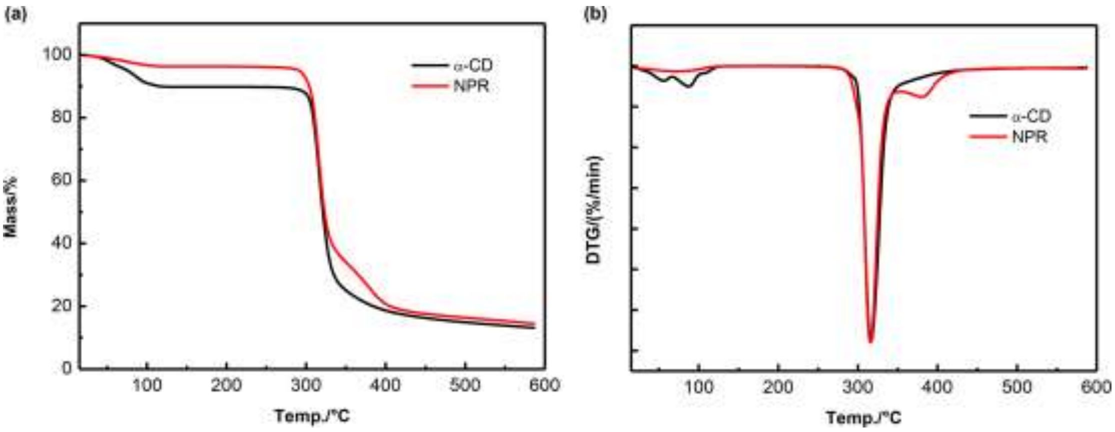


Figure S26. (a) Thermogravimetric analysis (TGA) curves and (b) Derivative thermogravimetry (DTG) of α-CD and NPR.

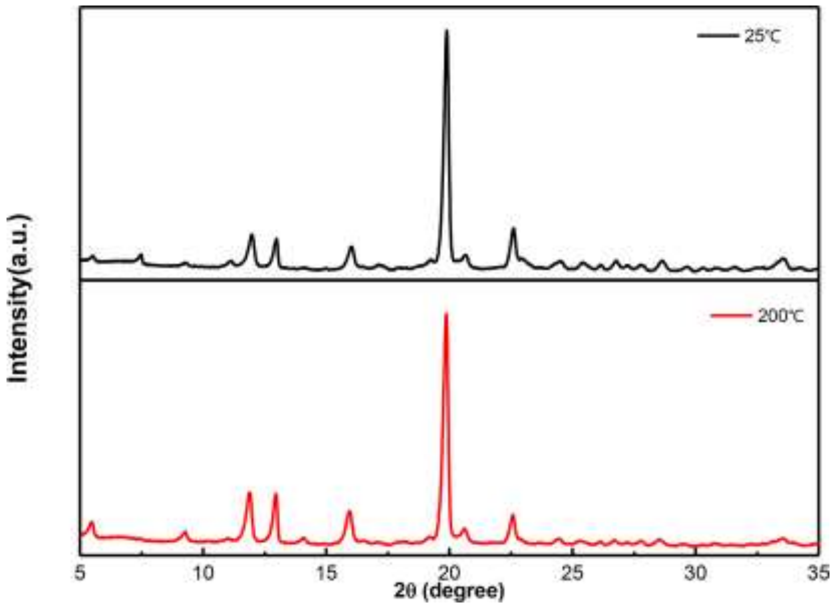


Figure S27. XRD spectra of NPR at different temperatures.

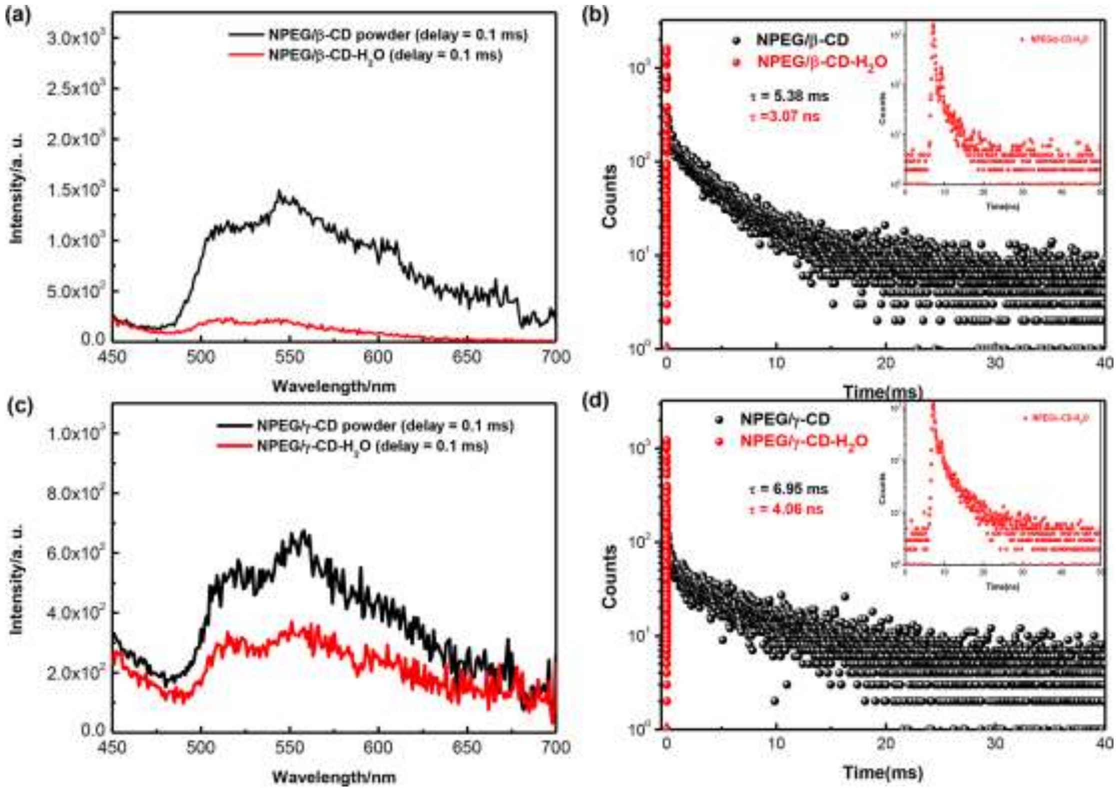


Figure S28. (a) The delayed spectra (b) Time-resolved photoluminescence decay spectra at 556 nm of NPEG/β-CD in the solid powder and in the 90 wt% $H_2O$ (Inset: Enlarged Time-resolved photoluminescence decay spectrum of NPEG/β-CD in the 90 wt% $H_2O$) in the 90 wt% $H_2O$. (c) The delayed spectra (d) Time-resolved photoluminescence decay spectra at 556 nm of NPEG/γ-CD in the solid powder and in the 90 wt% $H_2O$ (Inset: Enlarged Time-resolved photoluminescence decay

spectrum of NPEG/γ-CD in the 90 wt% $H_2O$).

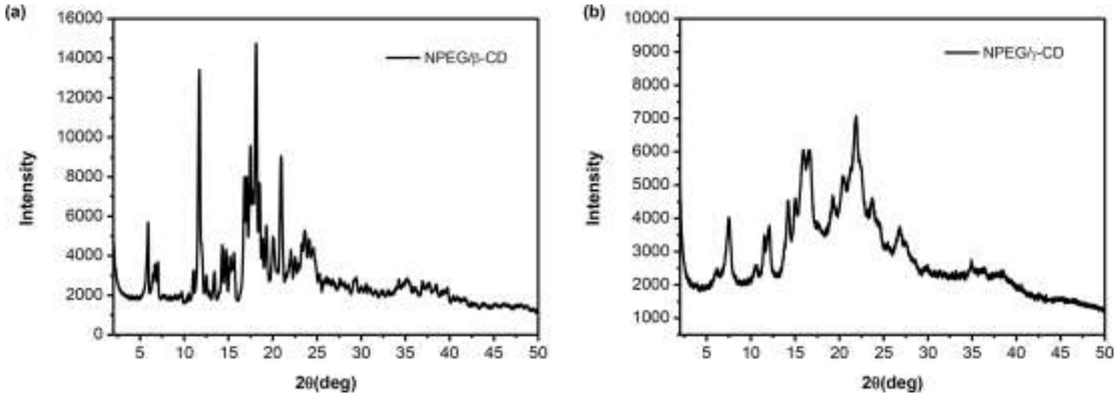


Figure S29. XRD spectrum of (a) NPEG/β-CD (b) NPEG/γ-CD.

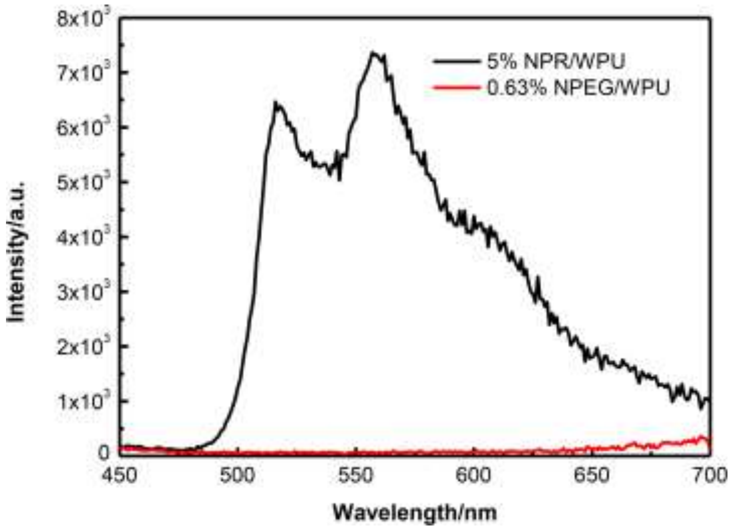


Figure S30. The phosphorescence emission spectra (delay = 0.1 ms) of 5% NPR/WPU and 0.63% NPEG/WPU (the same content of NPEG).

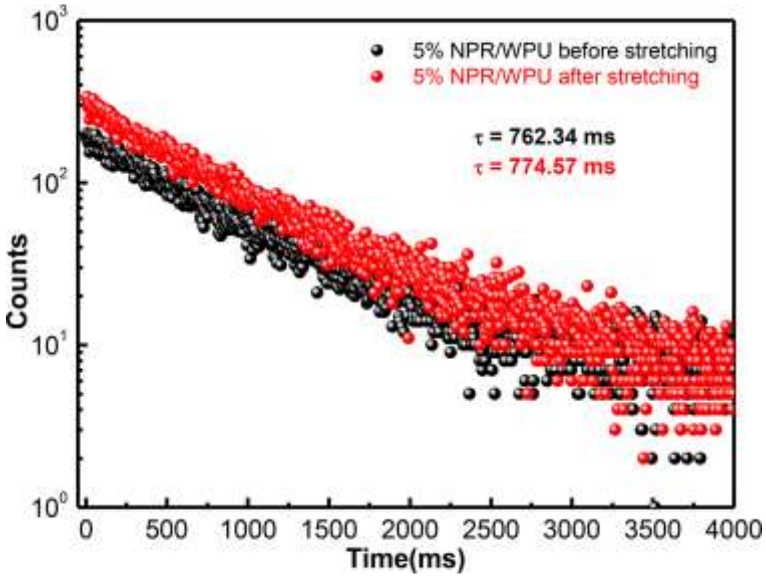


Figure S31. Time-resolved photoluminescence decay spectra of 5% NPR/WPU before

and after stretching.

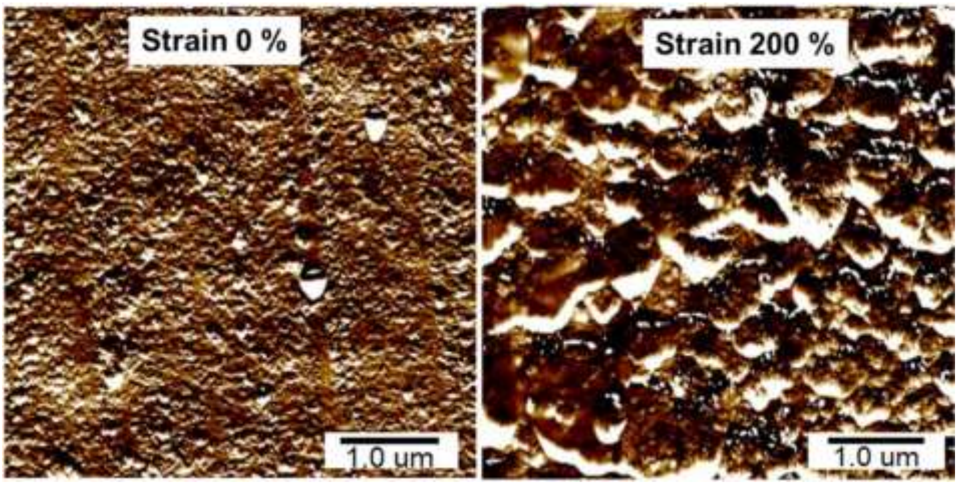


Figure S32. AFM phase diagram of 5% NPR/WPU films under 0% and 200% tensile strains.

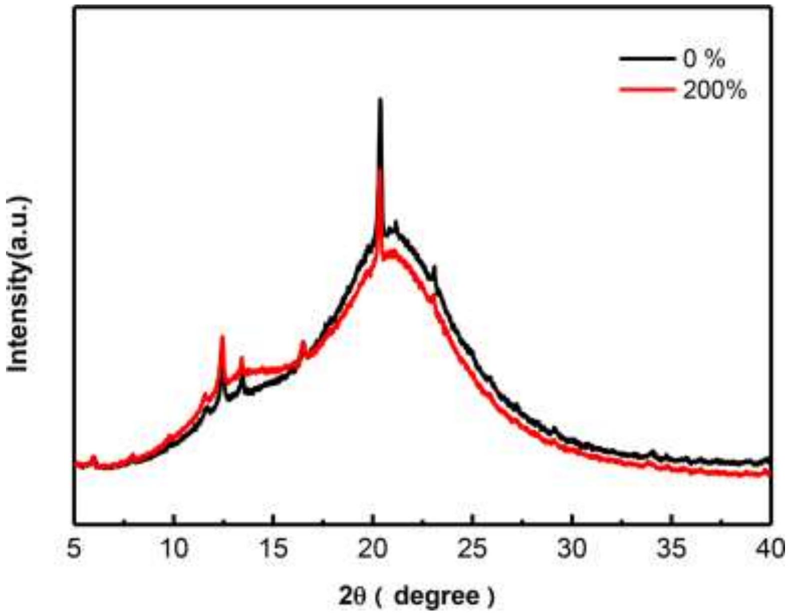


Figure S33. XRD of 5% NPR/WPU films under 0% and 200% tensile strains.

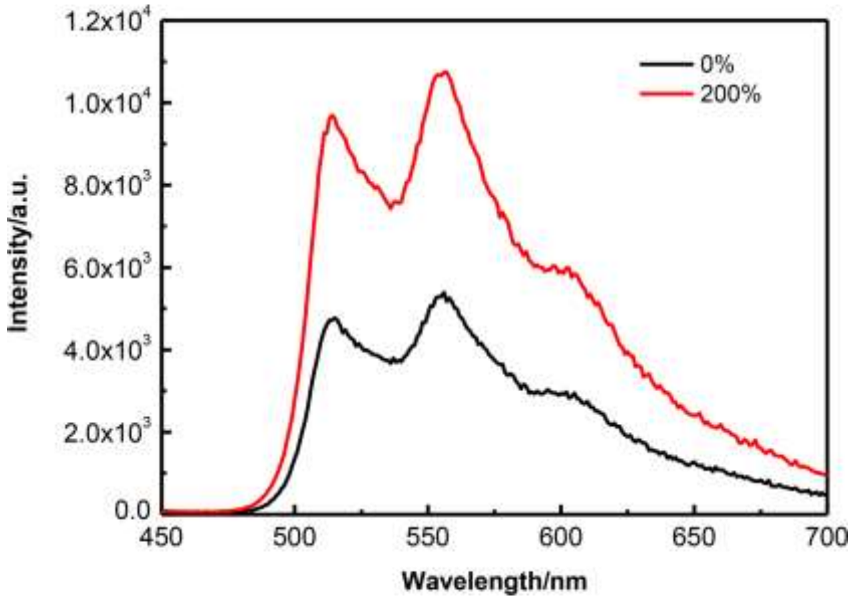


Figure S34. The delayed spectra (delay = 0.1 ms) of 5% NPR/WPU under different strain after 5 reciprocating cycles (maintain 0% strain for 8 hours per cycle).

## Supplemental references

[1] Y. Zhang, C. Zhang, Y. Chen, J. Yu, L. Chen, H. Zhang, X. Xu, Y. Liu, *Adv. Opt. Mater.* **2022,** *10*, 2102169.
